# Nunchaku: optimally partitioning data into piece-wise contiguous segments

**DOI:** 10.1093/bioinformatics/btad688

**Published:** 2023-11-15

**Authors:** Yu Huo, Hongpei Li, Xiao Wang, Xiaochen Du, Peter S Swain

**Affiliations:** Centre for Engineering Biology, University of Edinburgh, Edinburgh EH9 3BF, United Kingdom; School of Biological Sciences, University of Edinburgh, Edinburgh EH9 3BF, United Kingdom; School of Biological Sciences, University of Edinburgh, Edinburgh EH9 3BF, United Kingdom; School of Biological Sciences, University of Edinburgh, Edinburgh EH9 3BF, United Kingdom; School of Biological Sciences, University of Edinburgh, Edinburgh EH9 3BF, United Kingdom; Centre for Engineering Biology, University of Edinburgh, Edinburgh EH9 3BF, United Kingdom; School of Biological Sciences, University of Edinburgh, Edinburgh EH9 3BF, United Kingdom

## Abstract

**Motivation:**

When analyzing 1D time series, scientists are often interested in identifying regions where one variable depends linearly on the other. Typically, they use an *ad hoc* and therefore often subjective method to do so.

**Results:**

Here, we develop a statistically rigorous, Bayesian approach to infer the optimal partitioning of a dataset not only into contiguous piece-wise linear segments, but also into contiguous segments described by linear combinations of arbitrary basis functions. We therefore present a general solution to the problem of identifying discontinuous change points. Focusing on microbial growth, we use the algorithm to find the range of optical density where this density is linearly proportional to the number of cells and to automatically find the regions of exponential growth for both *Escherichia coli* and *Saccharomyces cerevisiae*. For budding yeast, we consequently are able to infer the Monod constant for growth on fructose. Our algorithm lends itself to automation and high throughput studies, increases reproducibility, and should facilitate data analyses for a broad range of scientists.

**Availability and implementation:**

The corresponding Python package, entitled Nunchaku, is available at PyPI: https://pypi.org/project/nunchaku.

## 1 Introduction

A common scientific problem is understanding the relationship between two variables. When the dependent variable, or some transformation of it, depends linearly on the independent variable, the underlying system linking the two often behaves more simply than generally. As a consequence, scientists commonly focus their efforts on identifying and understanding this linear regime.

A well-known example is the growth of a population of cells. In log phase, when the logarithm of the number of cells increases linearly with time, the total mass of every intracellular component grows exponentially and the mass per cell is approximately constant. Such steady-state conditions regularize growth; metabolic fluxes are balanced; and physiology simplifies, generating behaviours controlled by only a handful of variables (Scott and Hwa 2023).

Biologists therefore often wish to determine when growth is in log phase. Historically the approach has been to plot the logarithm of a variable correlating with the number of cells, such as optical density (OD), against time and to identify a linear region by eye (Monod 1949). Today this subjective technique is still used, with one scientist’s linear region not necessarily the same as another’s.

A challenge to developing objective approaches is identifying a suitable nonlinear model with which to compare the linear one. There is no general way to describe all relationships that we may observe. With a mechanistic understanding, we might generate a nonlinear description, but such an understanding is often lacking and, anyhow, may obviate the need to find linear regimes.

Here, we circumvent this problem by inferring the piece-wise linear description that best approximates an entire 1D time series. By doing so, we reframe the task to one of detecting change points—time points where the process generating the time series changes, a well-studied problem (Stephens 1994) with an established frequentist solution (Baranowski *et al.* 2019). We use a Bayesian approach, complementing others (Hutter 2007, Papastamoulis *et al.* 2019), and generalize by allowing each segment of data to be described by a linear combination of arbitrary basis functions, with straight lines being but one example. For a given set of basis functions, we compare the evidence for every possible piece-wise linear combination, found by marginalizing over all possible fits to all possible contiguous subdivisions of the data. For linear segments and for the optimal choice of segments, we provide statistics for each segment, allowing users to select straightforwardly the segment or segments of most interest. To illustrate our algorithm, we primarily discuss two examples: determining the range of OD of a liquid culture where the OD depends linearly on the number of cells and finding the exponential phases of microbial growth curves.

## 2 Materials and methods

### 2.1 Inferring contiguous regions using model comparison

Given 1D time-series data and a set of *K* basis functions, we wish to divide the data into the group of contiguous segments that is best characterized by piece-wise linear combinations of the basis functions. Irrespective of the data’s behaviour, we will always find such a group. Our approach answers two questions: how many piece-wise contiguous segments best describe the data given the basis functions and where the optimal segment boundaries lie.

Let us assume that we have observations, $\left(x_{j},y_{j}^{\left(r\right)}\right)$, where *j* runs from 1 to *N* and the *x_j_* are in ascending order; *r* indexes the *N_r_* replicates if any. We denote these observations collectively as *D*.

First, we consider whether we should divide the data into *M* or M′ segments, using Bayesian model comparison (MacKay 2003). Assuming equal prior probabilities, P(M)=P(M′), we write the Bayes’ factor as:


(1) $$\frac{P(M|D)}{P(M\prime |D)}=\frac{P(D|M)P(M)}{P(D|M\prime )P(M\prime )}=\frac{P(D|M)}{P(D|M\prime )},$$

and therefore we should determine the evidence P(D|M) for each *M*.

The evidence is a marginal likelihood. For *M* contiguous segments, there are *M–*1 unknown boundary points, which we denote as $n\equiv (n_{1},\ldots ,n_{M-1})$ with $n_{i}<n_{i+1}$. These points are integers and index an *x_j_*. The two remaining boundaries are the indices for the first and last *x* values: 1 and *N*. We assume that each segment contains a minimal number of data points $\ell_{\text{min}}$, so that $n_{i+1}\geq n_{i}+\ell_{\text{min}}$. The choice of $\ell_{\text{min}}$ depends on the type and number of basis functions: in general, $\ell_{\text{min}}\geq K$.

The evidence is a sum over all potential ***n***:


(2) $$\begin{array}{c} P(D|M)=\sum_{n}P(D|n,M)P(n|M)\\ =f(N,M,\ell_{\text{min}})\sum_{n}P(D|n,M) \end{array}$$

where we use that any permissible *n_i_* is equally likely as any other to write the prior P(n|M) as a function of *N*, *M*, and $\ell_{\text{min}}$. Specifically, this bounded uniform prior is the reciprocal of the number of possible ***n***, which satisfy


(3) $$n_{1}\geq \ell_{\text{min}},\;n_{2}\geq n_{1}+\ell_{\text{min}},\ldots ,n_{M-1}\geq N-\ell_{\text{min}}.$$

for a given *M* and $\ell_{\text{min}}$. We therefore have:


(4) $$\begin{array}{c} P(n|M)\;=\;[\sum_{n_{1}\;=\;\ell_{\text{min}}}^{N-(M-1)\ell_{\text{min}}}\times \sum_{n_{2}\;=\;n_{1}+\ell_{\text{min}}}^{N-(M-2)\ell_{\text{min}}}\times \cdots \\ \times \sum_{n_{M-1}\;=\;n_{M-2}+\ell_{\text{min}}}^{N-\ell_{\text{min}}}1{]}^{-1}\\ \;=\;f(N,M,\ell_{\text{min}}). \end{array}$$

Second, for a given *M* and ***n***, we fit the data to *M* different linear combinations of the basis functions, one for each segment, with each combination independent of the other. The linear combination ending near the data points indexed by *n_i_* and $n_{i+1}$ depends only on the data indexed by the indices $n_{i}+1$ and $n_{i+1}$ inclusively, denoted *D_i_*, and this data does not determine any other linear combination. Therefore, mathematically,


(5) $$\begin{array}{c} P\left(D|n,M\right)=P(D_{1}|1,n_{1})\times P(D_{2}|n_{1}+1,n_{2})\times \cdots \\ \times P(D_{M}|n_{M-1}+1,N) \end{array}$$

where $P(D_{i}|n_{i}+1,n_{i+1})$ is the likelihood of a linear combination of the basis functions describing the data indexed by $n_{i}+1$ to $n_{i+1}$.

#### 2.1.1 Finding $P(D|n_{,}M)$

For each segment of the data, we consider the *K* basis functions, each individually denoted $\phi_{k}(x)$ and collectively ϕ(x), and correspondingly *K* coefficients, each denoted *m_k_*. If fitting lines, we have two basis functions: $\phi_{1}=1$ and $\phi_{2}=x$, and two *m_k_* where *m*_1_ determines the line’s *y*-intercept and *m*_2_ its gradient. We then set $\ell_{\text{min}}=3$ so that there are sufficient data points in each segment to define a line.

We let $P(y_{j}|x_{j},m)$ describe how a data point *y_j_* at *x_j_* deviates from the linear combination of basis functions and assume that this deviation is independent of the deviations of other data points.

For the *i*th segment, we then have


(6) $$\begin{array}{c} P\left(D_{i}|n_{i}+1,n_{i+1}\right)=\int dm\;P(m)\prod_{r=1}^{N_{r}}\prod_{j=n_{i}+1}^{n_{i+1}}P(y_{j}^{\left(r\right)}|x_{j},m)\\ =P(m)\int dm\;\prod_{r=1}^{N_{r}}\prod_{j=n_{i}+1}^{n_{i+1}}P(y_{j}^{\left(r\right)}|x_{j},m) \end{array}$$

assuming the prior P(m) is a constant, with each *m_k_* independently and uniformly distributed in some bounded region so that


(7) $$P(m)=\{\begin{array}{cc} \frac{1}{\left(m_{1}^{\text{max}}-m_{1}^{\text{min}})\cdots (m_{K}^{\text{max}}-m_{K}^{\text{min}}\right)} & \text{for}\;m_{i}\in [m_{i}^{\text{min}},m_{i}^{\text{max}}]\\ 0\; & \text{otherwise} \end{array}$$

for fixed $m_{k}^{\text{min}}$ and $m_{k}^{\text{max}}$ for all *k*.

#### 2.1.2 Marginalizing P(D|n,M)

Using Equation (5), we factorize the sum in Equation (2):


(8) $$\begin{array}{c} \sum_{n}P(D|n,M)=\sum_{n_{1}=\ell_{\text{min}}}^{N-(M-1)\ell_{\text{min}}}P(D_{1}|1,n_{1})\\ \times \sum_{n_{2}=n_{1}+\ell_{\text{min}}}^{N-(M-2)\ell_{\text{min}}}P(D_{2}|n_{1}+1,n_{2})\times \cdots \\ \times \sum_{n_{M-2}=n_{M-3}+\ell_{\text{min}}}^{N-2\ell_{\text{min}}}P(D_{M-2}|n_{M-3}+1,n_{M-2})\\ \times \sum_{n_{M-1}=n_{M-2}+\ell_{\text{min}}}^{N-\ell_{\text{min}}}P(D_{M-1}|n_{M-2}+1,n_{M-1})\\ \times P(D_{M}|n_{M-1},N) \end{array}$$

and use the method of variable elimination (Zhang and Poole 1996) to evaluate these sums. First we perform the rightmost one, over $n_{M-1}$, to generate a function of $n_{M-2}$. We then perform the next rightmost sum, over $n_{M-2}$, of this function and the next term in Equation (8), which generates a function of $n_{M-3}$. We repeat this process until we reach the leftmost sum over *n*_1_, enabling $O(MN^{2})$ operations in total instead of $O(N^{M})$. We evaluate Equation (4) similarly.

All that remains is to determine $P(D_{i}|n_{i}+1,n_{i+1})$ so that we can find P(D|M) via Equation (2) and Equation (8).

#### 2.1.3 Finding $P(D_{i}|n_{i}+1,n_{i+1})$ for known measurement error

To proceed, we assume that $P(y_{j}|x_{j},m)$ is a normal distribution with mean $\phi {\left(x_{j}\right)}^{T}m$, or equivalently $\sum_{k}m_{k}\phi_{k}(x_{j})$, and a standard deviation *σ_j_*. If we know the *σ_j_*, e.g. by approximating each by the corresponding measurement error, then Equation (6), the likelihood of a linear combination describing the data indexed by $n_{i}+1$ to $n_{i+1}$, becomes


(9) $$\begin{array}{c} P(D|n_{i}+1,n_{i+1},\sigma )=P(m)\prod_{j=n_{i}+1}^{n_{i+1}}{\left(\sqrt{2\pi }\sigma_{j}\right)}^{-N_{r}}\\ \times \int dm\;\text{exp}\;\left[-\sum_{r=1}^{N_{r}}\sum_{j=n_{i}+1}^{n_{i+1}}\frac{{\left[y_{j}^{\left(r\right)}-\phi {\left(x_{j}\right)}^{T}m\right]}^{2}}{2\sigma_{j}^{2}}\right]. \end{array}$$

To evaluate the integral, we extend it to infinite range for all *m_k_—*a suitable approximation because we expect the integrand to be strongly peaked at the most likely values of each *m_k_* (MacKay 2003). We can then perform the integration analytically.

Consider data with a single replicate. Define $\ell_{i}=n_{i+1}-n_{i}$ to be the number of *x* values in the *i*th segment and $z^{\left(i\right)}$ to be a vector with components $y_{j}/\sigma_{j}$, with the superscript *i* used to denote the *i*th segment. Let Φ(X) be the $K\times \ell_{i}$ matrix with components $\Phi_{kj}=\phi_{k}(x_{j})/\sigma_{j}$, and further defining


(10) $$\begin{array}{ccc} A^{\left(i\right)}=\Phi \Phi^{T} & ; & {\bar{m}}^{\left(i\right)}={\left(A^{\left(i\right)}\right)}^{-1}\Phi z^{\left(i\right)} \end{array}$$

so that $A_{kk\prime }^{\left(i\right)}=\sum_{j}\phi_{k}(x_{j})\phi_{k\prime }(x_{j})$. The matrix $A^{\left(i\right)}$ is a symmetric *K *×* K* matrix, which is invertible when the basis functions $\phi_{k}$ are linearly independent and when $\ell_{i}\geq K$. Then standard algebra gives


(11) $$\sum_{j=n_{i}+1}^{n_{i+1}}\frac{{\left[y_{j}-\phi {\left(x_{j}\right)}^{T}m\right]}^{2}}{2\sigma_{j}^{2}}=\frac{1}{2}{\left(m-{\bar{m}}^{\left(i\right)}\right)}^{T}A^{\left(i\right)}(m-{\bar{m}}^{\left(i\right)})+U^{\left(i\right)}$$

where


(12) $$2U^{\left(i\right)}={\left(z^{\left(i\right)}\right)}^{T}z^{\left(i\right)}-{\left({\bar{m}}^{\left(i\right)}\right)}^{T}A^{\left(i\right)}{\bar{m}}^{\left(i\right)}.$$

Using Equation (11) and the results for integrating multivariate Gaussian distributions (MacKay 2003), we have that


(13) $$\begin{array}{c} \int dm\;\text{exp}\;\left[-\sum_{j=n_{i}+1}^{n_{i+1}}\frac{{\left[y_{j}-\phi {\left(x_{j}\right)}^{T}m\right]}^{2}}{2\sigma_{j}^{2}}\right]={\left(2\pi \right)}^{\frac{K}{2}}{\left(\text{det}A^{\left(i\right)}\right)}^{-\frac{1}{2}}\\ \times e^{-U^{\left(i\right)}}. \end{array}$$

If we are fitting straight lines with *K *=* *2 and $\phi_{1}=1$ and $\phi_{2}=x$, then it is useful to define (Hinrichsen *et al.* 2017)


(14) $$\begin{array}{ccccccc} T_{1} & = & \sum_{j}\frac{y_{j}^{2}}{2\sigma_{j}^{2}} & ; & T_{2} & = & \sum_{j}\frac{x_{j}^{2}}{2\sigma_{j}^{2}}\\ T_{3} & = & \sum_{j}\frac{1}{2\sigma_{j}^{2}} & ; & T_{4} & = & \sum_{j}\frac{y_{j}}{\sigma_{j}^{2}}\\ T_{5} & = & \sum_{j}\frac{x_{j}y_{j}}{\sigma_{j}^{2}} & ; & T_{6} & = & \sum_{j}\frac{x_{j}}{\sigma_{j}^{2}} \end{array}$$

with *j* running from $n_{i}+1$ to $n_{i+1}$. Using these definitions,


(15) $$\begin{array}{c} \begin{array}{cccc} A^{\left(i\right)}=\left(\begin{array}{cc} 2T_{3} & T_{6}\\ T_{6} & 2T_{2} \end{array}\right) & ; & {\bar{m}}^{\left(i\right)}=\left(\begin{array}{c} \frac{2T_{2}T_{4}-T_{5}T_{6}}{4T_{2}T_{3}-T_{6}^{2}}\\ \frac{2T_{3}T_{5}-T_{4}T_{6}}{4T_{2}T_{3}-T_{6}^{2}} \end{array}\right) & \end{array}\\ U^{\left(i\right)}=T_{1}-\frac{T_{2}T_{4}^{2}+T_{3}T_{5}^{2}-T_{4}T_{5}T_{6}}{4T_{2}T_{3}-T_{6}^{2}} \end{array}$$

and the integral becomes $(2\pi ){\left(4T_{2}T_{3}-T_{6}^{2}\right)}^{-\frac{1}{2}}e^{-U^{\left(i\right)}}$.

With more than one replicate, ***z*** runs over all *y* in all replicates, with the replicates arranged contiguously, and is of length $N_{r}\ell_{i}$; Φ has rows of length $N_{r}\ell_{i}$ with $x_{n_{i}+1}$ to $x_{n_{i+1}}$ repeated *N_r_* times in each row to match the corresponding *y* values. For the linear case, the sums in Equation (14) are over both *j* and the number of replicates, so that *T*_1_, e.g., becomes $\sum_{j,r}\frac{{\left(y_{j}^{\left(r\right)}\right)}^{2}}{2\sigma_{j}^{2}}$.

Returning to Equation (9), we find


(16) $$\begin{array}{c} P(D_{i}|n_{i}+1,n_{i+1},\sigma )=P(m)\left(\prod_{j=n_{i}+1}^{n_{i+1}}{\left(\sqrt{2\pi }\sigma_{j}\right)}^{-N_{r}}\right)\\ \times {\left(2\pi \right)}^{\frac{K}{2}}{\left(\text{det}A^{\left(i\right)}\right)}^{-\frac{1}{2}}e^{-U^{\left(i\right)}} \end{array}$$

with the help of Equation (13). For this approximation to be valid, we require that the strongly peaked region in ***m*** space is within the *a priori* range for ***m***. The area under the integrand in Equation (13) is proportional to the square root of $\text{det}A^{\left(i\right)}$, and the prior range of ***m*** must be large enough to contain this area. Using Equation (7), we need


(17) $${\left(\text{det}A^{\left(i\right)}\right)}^{\frac{1}{2}}\times P(m)\ll 1.$$

#### 2.1.4 Finding the boundary points

After determining the optimal number of segments into which to divide the data from Equaion (1), we next find their boundary points. Using Bayes’ theorem, the posterior for *n* is


(18) $$P(n|D,M,\sigma )=\frac{P(D|n,M,\sigma )P(n|M)}{P(D|M,\sigma )}$$

which we evaluate using Equations (2, 4, and 5). We use the mean posterior value of *n_i_* to estimate the optimal *n_i_*:


(19) $$\begin{array}{c} E[n_{i}]=\sum_{n}n_{i}P(n|D,M,\sigma )\\ =\frac{P(n|M)}{P(D|M,\sigma )}\sum_{n}n_{i}P(D|1,n_{1},\sigma )\cdots P(D|n_{M-1},N,\sigma ) \end{array}$$

which we sum following Equation (8). The posterior variance, $\text{Var}[n_{i}]$, determines the error in this estimate, which we find similarly.

#### 2.1.5 Finding P(D|M) for unknown measurement error

If the *σ_j_* are unknown, we assume the same constant *σ* for all *j* with a uniform prior probability between $\left[\sigma_{\text{min}},\sigma_{\text{max}}\right]$ (Gelman 2006). Equation (2) then becomes


(20) $$\begin{array}{c} P(D|M)=f(N,M,\ell_{\text{min}})\sum_{n}P(D|n,M)\\ =f(N,M,\ell_{\text{min}})P(\sigma )\sum_{n}\int_{\sigma_{\text{min}}}^{\sigma_{\text{max}}}d\sigma P(D|n,M,\sigma ). \end{array}$$

The constant $P(\sigma )=1/(\sigma_{\text{max}}-\sigma_{\text{min}})$ will cancel in Equation (1) when we compare the evidence for different *M*.

Using the equivalent of Equations (9 and 13), we find that


(21) $$\begin{array}{c} P(D_{i}|n_{i}+1,n_{i+1},\sigma )=P(m){\left(\sqrt{2\pi }\sigma \right)}^{-N_{r}\ell_{i}+K}\\ \times {\left(\text{det}A^{\left(i\right)}\right)}^{-\frac{1}{2}}\;\text{exp}\;\left[-\frac{U^{\left(i\right)}}{\sigma^{2}}\right] \end{array}$$

where we now explicitly follow *σ* and so set the *σ_j_* in Equation (10) to unity, making *z_i_* = *y_i_* and $\Phi_{kj}=\phi_{k}(x_{j})$. Similarly for the linear case, the *σ_j_* become unity in Equation (14).

Consequently,


(22) $$\begin{array}{c} P(D|n,M,\sigma )=P(D_{1}|1,n_{1},\sigma )\times P(D_{2}|n_{1}+1,n_{2},\sigma )\times \cdots \\ \times \;P(D_{M}|n_{M-1}+1,N,\sigma )\\ =P{\left(m\right)}^{M}{\left(\sqrt{2\pi }\sigma \right)}^{-N_{r}N+MK}\prod_{i=1}^{M}{\left(\text{det}A^{\left(i\right)}\right)}^{-\frac{1}{2}}\\ \times \;\text{exp}\;\left(-\frac{\sum_{i=1}^{M}U^{\left(i\right)}}{\sigma^{2}}\right). \end{array}$$

Although with Equation (22) it is possible to approximate analytically the integral over *σ* in Equation (20) by extending the range of the integrand to (0,∞), the resulting expression prevents us from summing over ***n*** using variable elimination. Instead, we swap the sum and the integral to write


(23) $$P(D|M)=f(N,M,\ell_{\text{min}})P(\sigma )\int_{\sigma_{\text{min}}}^{\sigma_{\text{max}}}d\sigma \sum_{n}P(D|n,M,\sigma )$$

and numerically evaluate, using variable elimination to sum over ***n*** in Equation (23) for each *σ* chosen by the integration algorithm.

We find the expected boundary points via Equation (19), again numerically integrating over *σ*.

**Performing the integration:** To stabilize the numerical integration, we scale the integrand of Equation (23) by its value at the most likely value of *σ*, making the integrand nearly always less than one and preventing overflow. We use expectation-maximization (EM) to estimate the most likely *σ* for a given *M*. The EM algorithm finds the *σ* that maximizes P(D|M,σ) (Bishop 2006). We guess a value of *σ*, *σ_o_* say, and find $P(n|D,\sigma_{o},M)$ from Equation (18). To update *σ_o_*, we maximize $Q(\sigma ,\sigma_{o})$ with respect to *σ*, where


(24) $$\begin{array}{c} Q(\sigma ,\sigma_{o})=\sum_{n}P(n|D,M,\sigma_{o})\;\text{log}\;P(D,n|M,\sigma )\\ =E[\text{log}\;P(D|n,M,\sigma )+\text{log}\;P(n|M,\sigma )]\\ =E[\text{log}\;P(D|n,M,\sigma )+\text{log}\;f(N,M,\ell_{\text{min}})] \end{array}$$

with the expectations taken over $P(n|D,M,\sigma_{o})$. Expanding Equation (24) using Equation (22), there are only two terms that depend on *σ*, and we can differentiate to find the updated $\sigma =\sigma_{n}$:


(25) $$\sigma_{n}^{2}=\frac{2}{N_{r}N-MK}\sum_{i=1}^{M}E[U_{i}].$$

We use the equivalent of Equation (19) with $\sigma =\sigma_{o}$ to evaluate these expectations and iterate until the value of *σ* converges.

#### 2.1.6 Implementation

For basis functions that generate lines, we compare the different linear segments by calculating the gradient, intercept, and the coefficient of determination *R*^2^ of the line maximizing the likelihood for each segment. The user can then select a desired segment, such as the one with the largest gradient.

The algorithm requires the *a priori* bounded region of ***m*** in Equation (7). Again specializing to straight lines, the prior specifies the range of the intercept *m*_1_ and the gradient *m*_2_: $\left[m_{1}^{\text{min}},m_{1}^{\text{max}}\right]$ and $\left[m_{2}^{\text{min}},m_{2}^{\text{max}}\right]$. The user can either provide both ranges or only the range of *m*_2_ or give the maximal range of *y* possible in the experiment, $\left[y_{\text{min}},y_{\text{max}}\right]$. If the user provides only the range of *m*_2_, we estimate $m_{1}^{\text{min}}$ as $\min\left(-m_{2}^{\text{max}}x_{\text{max}},m_{2}^{\text{min}}x_{\text{min}}\right)$ and $m_{1}^{\text{max}}$ as $\max\left(-m_{2}^{\text{min}}x_{\text{max}},m_{2}^{\text{max}}x_{\text{min}}\right)$. If the user provides the range of *y*, we estimate the range of *m*_2_ as $\left[-g_{\text{max}},g_{\text{max}}\right]$, with $g_{\text{max}}=(y_{\text{max}}-y_{\text{min}})/\Delta x_{\text{min}}$ and $\Delta x_{\text{min}}$ being the smallest difference between two neighbouring *x* values.

#### 2.1.7 Availability

We coded the algorithm as a Python package available at https://pypi.org/project/nunchaku and via pip. We have also embedded nunchaku into our omniplate software for analyzing plate-reader data (Montaño-Gutierrez *et al.* 2022).

#### 2.1.8 Generating and testing with synthetic data

**Figure 1.**
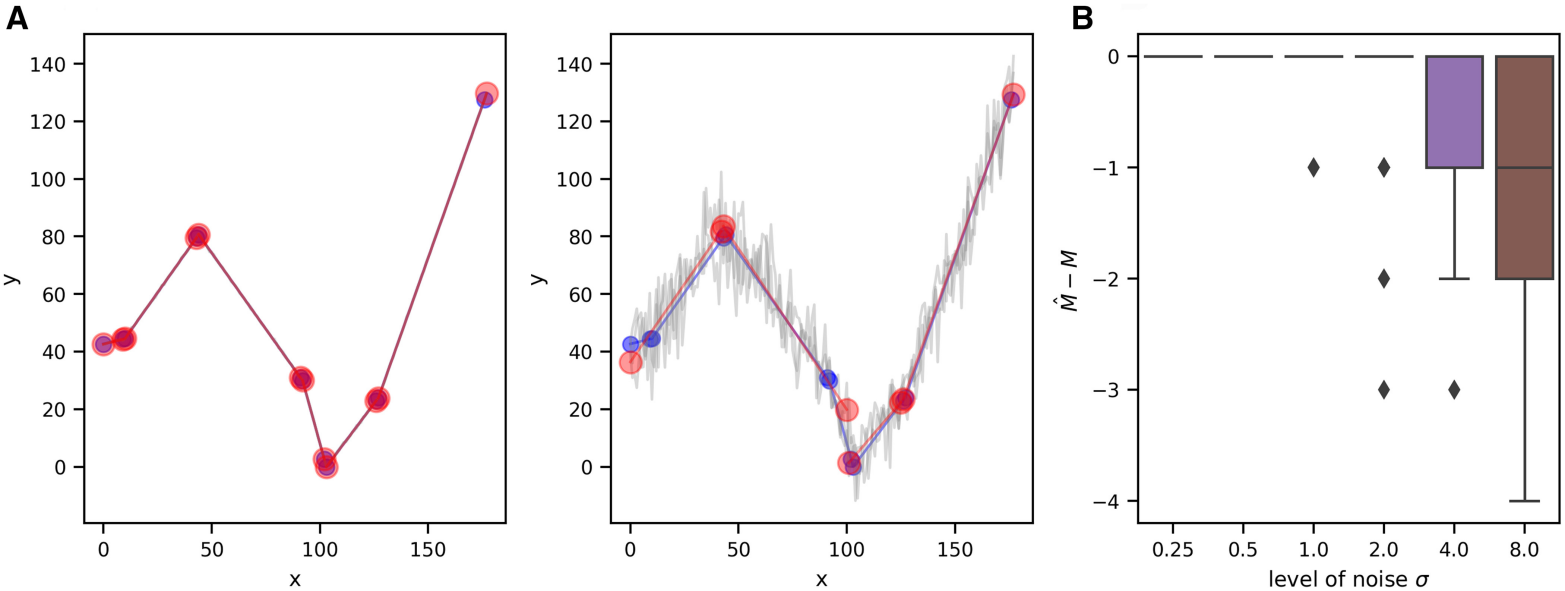
The nunchaku algorithm correctly predicts the number of linear segments in synthetic data when the measurement noise is not too high. (A) Example synthetic datasets with the ground truth in blue (small circles) and the triplicate data in light grey. The large red circles are the predicted boundaries of each linear segment with the best-fit line in red. Left: with a measurement error of 0.25, the predictions overlap the data; Right: with a measurement error of 8, the predictions miss some segments, which the noise obscures. As a prior, we specify only that the gradient of each line lies between [−25,25]. For this data, a measurement error of 0.25 is 0.5% of the mean of *y* and an error of 8 is almost 15%. (B) The algorithm underestimates the number of linear segments only once the magnitude of the measurement noise becomes sufficiently high. The actual number of segments is *M*; the estimated number is $\hat{M}$.

**Figure 2.**
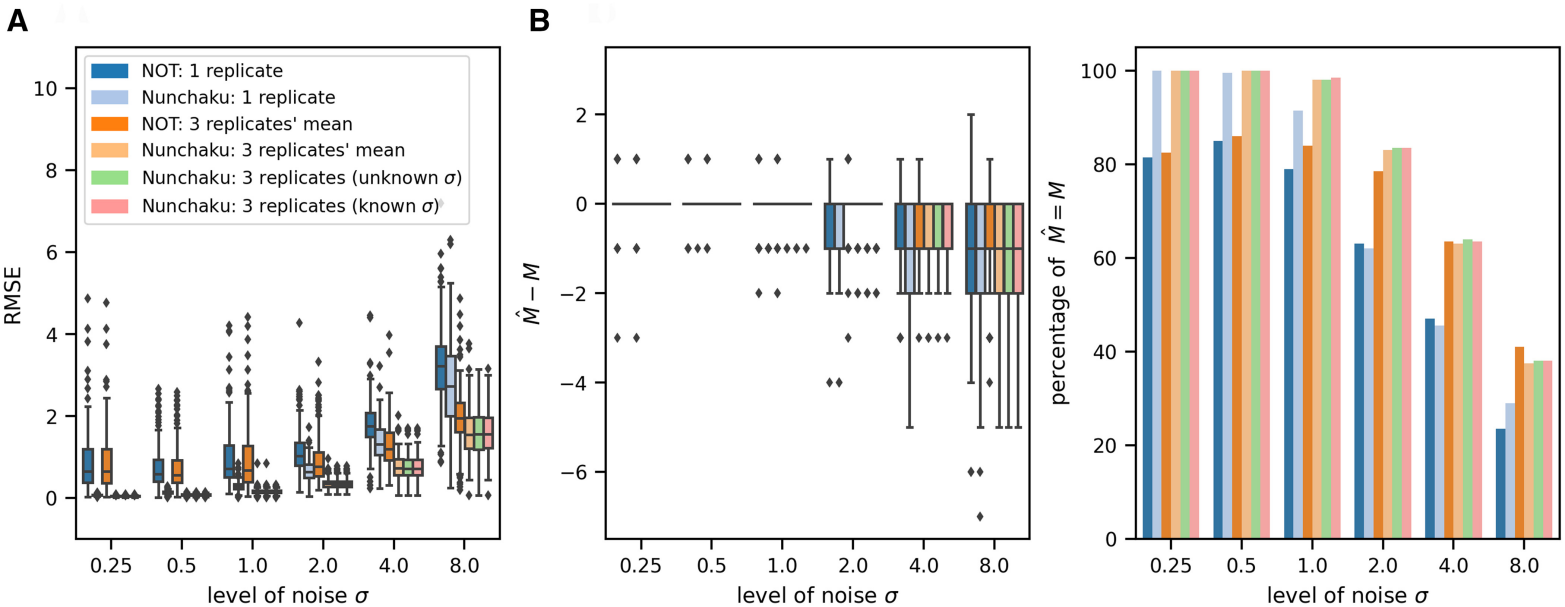
Nunchaku performs as well as or better than the NOT algorithm (Baranowski *et al.* 2019). This algorithm only supports input of one *y* value for each *x* value: we therefore input either one replicate or the mean of three replicates. The data are generated similarly to that in Fig. 1 (Section 2). As a prior for nunchaku, we specify that the gradient of each line lies between [−25,25]. (A) The root mean squared error (RMSE) between the ground truth and the best-fit lines. (B) The difference between the predicted number of segments $\hat{M}$ and the ground truth *M* (left) and the percentage of correct predictions of *M* with $\hat{M}=M$ (right).

To test our method, we generated a piece-wise linear function *f*(*x*) with 1≤M≤10 continuous linear segments, each having between 10 and 50 data points and with a unit distance, Δx=1, between data points. We sampled *θ*, the angle between each segment and the *x*-axis, from a uniform distribution on the interval $\left[-{\text{tan}\;}^{-1}(20),{\;\text{tan}\;}^{-1}(20)\right]$, so that the gradient, tan θ, lies between [−20,20]. Furthermore we ensured that the difference in *θ* between neighbouring segments is larger than a fixed minimum, *θ*_0_. We added Gaussian noise, $\epsilon ∼\text{Normal}(0,\sigma^{2})$, to give three replicates of y=f(x)+ϵ. We generated 3600 synthetic datasets in total, a combination of 200 different piece-wise linear functions *f*(*x*), three values of *θ*_0_, and six values of *σ*. In Figs 1 and 2, $\theta_{0}=10^{{}^{\circ}}$.

### 2.2 Experimental methods

We used a prototrophic strain of *Saccharomyces cerevisiae* (FY4), precultured in synthetic complete (SC) medium with 2% (w/v) sodium pyruvate in a 30°C shaking incubator at 180 rpm for two days. Before the experiment, we diluted the cells 6-fold and let them grow for six hours. After washing the cells twice with fresh minimal media (Verduyn *et al.* 1992), we inoculated them into minimal media with different concentrations of fructose on a 96-well microplate. The liquid volume of each well was 200 μl.

For *Escherichia coli*, we precultured cells in 3 ml liquid Luria broth (LB) with one colony from a fresh plate and grew aerobically to log phase (6 h) at 37°C with 250 rpm shaking. We then inoculated 3 μl culture into 147 μl fresh LB medium per well on a 96-well microplate.

We used either a Tecan Infinite M200 Pro or F200 plate reader at 30°C for *S.cerevisiae* and 37°C for *E.coli* with linear shaking at amplitude 6 mm. Measurements of absorbance at 600 nm, OD_600_, were taken every 10 min.

Data were analyzed using the omniplate software (Montaño-Gutierrez *et al.* 2022).

### 2.3 Fitting Monod’s equation

After estimating the specific growth rate *λ* at each concentration of fructose *s*, we have a dataset $D\equiv \{(\lambda_{i},s_{i})\}$ with 38 data points. We use Bayesian inference to estimate the constants $\lambda_{\text{max}}$ and *K_M_* of Monod’s equation. Assuming a Gaussian measurement error of $\lambda_{\text{max}}$ with a standard deviation *σ* and independent measurements, the likelihood


(26) $$\begin{array}{c} P(D|\lambda_{\text{max}},K_{M},\sigma )={\left(\sqrt{2\pi }\sigma \right)}^{-N}\\ \times \prod_{i=1}^{N}\;\text{exp}\;\left[-\frac{{\left(\lambda_{i}-\lambda_{\text{max}}\frac{s_{i}}{K_{M}+s_{i}}\right)}^{2}}{2\sigma^{2}}\right]. \end{array}$$

To marginalize over *σ*, we assume P(σ)∝1/σ, so that


(27) $$\begin{array}{c} P(D|\lambda_{\text{max}},K_{M})\propto \int_{0}^{\infty }d\sigma P(D|\lambda_{\text{max}},K_{M},\sigma )P(\sigma )\\ \propto {\left[\sum_{i=1}^{N}{\left(\lambda_{i}-\lambda_{\text{max}}\frac{s_{i}}{K_{M}+s_{i}}\right)}^{2}\right]}^{-\frac{N}{2}}. \end{array}$$

We further assume that the prior $P(\lambda_{\text{max}},K_{M})$ is uniform, and so the posterior probability $\lambda_{\text{max}}$ and *K_M_* is proportional to the likelihood, Equation (27). We therefore maximize the likelihood with respect to $\lambda_{\text{max}}$ and *K_M_* using the BFGS algorithm. We estimate the errors in these inferences using the diagonal elements of the Hessian matrix $-\nabla \nabla \;\text{log}\;P(D|\lambda_{\text{max}},K_{M})$ evaluated at the maximum of the likelihood (MacKay 2003).

## 3 Results

### 3.1 Approximating data with a piece-wise linear model

Although our goal is to allow scientists to choose objectively the segment of their data that is ‘most’ linear, we adopt a general methodology and allow the data to be described by linear combinations of arbitrary basis functions. For straight lines, there are two basis functions, $\phi_{1}(x)=1$ and $\phi_{2}(x)=x$, but datasets may require higher order polynomials or even Gaussian or sigmoid functions (Bishop 2006).

For a 1D time series and a given set of basis functions, we will infer the optimal piece-wise description—the number of contiguous segments into which we should divide the data, where the boundaries of each of those segments should be, and the best-fit linear combination of basis functions for each segment. Deciding which of these segments is then most appropriate for the task in hand is unavoidably subjective. It is straightforward, however, to compare different segments by comparing properties of their best-fit linear combinations. For lines, these properties include their gradients and *R*^2^ value—how much of the variance of the dependent variable is explained by the independent one (Moses 2017).

We use a Bayesian approach to infer the best piece-wise description and assume only that the data of each segment is normally distributed around a linear combination of the basis functions (Section 2). To proceed analytically we marginalize over all coefficients constituting the linear combination for each segment using a mild approximation and choose the optimal number of segments by comparing marginal likelihoods. The data points bounding each segment are then estimated by the means of their posterior distribution. We consider the case with known measurement error separately from an unknown one and call our algorithm nunchaku.

### 3.2 Verifying our approach

To verify our methodology (Section 2), we first focused on identifying linear regions. We generated synthetic data using piece-wise linear functions, where we know the number of segments and their gradients, added Gaussian noise, and then inferred from this data the optimal number of segments and the gradients of the best-fit lines, assuming that we know the magnitude of the measurement noise (Fig. 1A).

The algorithm predicts correctly the number of segments when the noise in the data is sufficiently low (Fig. 1B and Supplementary Fig. S1), but underestimates this number when the noise is larger. Such noise tends to blur two neighbouring segments so they seem one, rather than cause a single segment to appear as two or more. Similarly, if we decrease the angle between neighbouring segments, the noise is more likely to make two neighbouring segments appear contiguous, and the algorithm’s accuracy falls (Supplementary Fig. S1).

We confirmed that the algorithm also correctly predicts the underlying piece-wise linear functions, and hence the gradient of the lines generating the data in the segments (Supplementary Fig. S1). As expected, this accuracy falls too with more noisy data.

When the measurement error is unknown, the results are similar (Supplementary Fig. S1), but the algorithm is slower because we numerically integrate over all possible magnitudes of this measurement error. We also confirmed that the algorithm’s performance is robust to broad choices of the prior distribution (Supplementary Fig. S2).

We next compared our methodology to the Narrowest-Over-Threshold (NOT) algorithm (Baranowski *et al.* 2019), a state-of-the-art frequentist approach. Whether we consider the root mean square error between the best-fit lines and the ground truth (Fig. 2A) or the predicted number of segments (Fig. 2B), our algorithm consistently performs as well as or better (see also Supplementary Fig. S3). This greater accuracy however comes at the expense of speed: the NOT algorithm is faster than our implementation of nunchaku.

Finally, we demonstrated that nunchaku works with other basis functions, including constant functions, third-order polynomials, and sines (Supplementary Fig. S4).

### 3.3 Application 1: finding the range of OD that increases linearly with cell number

**Figure 3.**
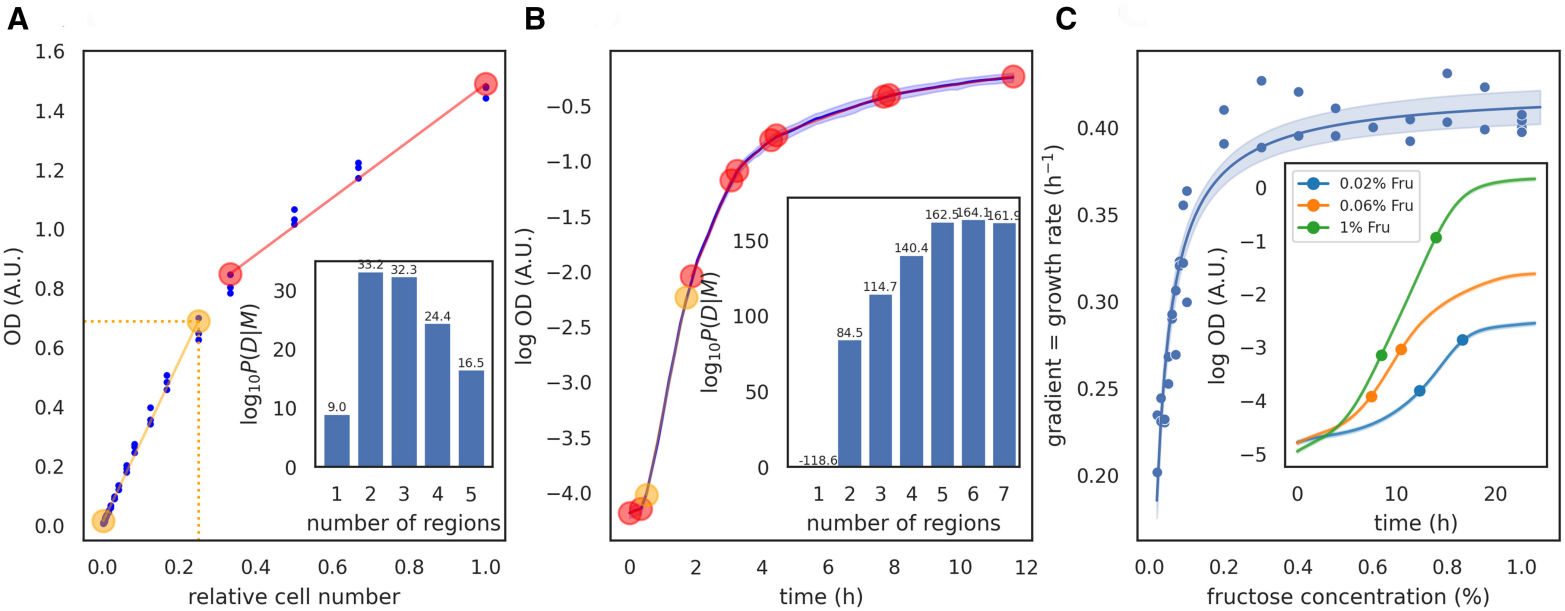
The nunchaku algorithm gives intuitive results when applied to biological data. (A) The calibration curve for plate-reader measurements of the OD of *S.cerevisiae*, found by diluting an overnight culture in 2% fructose, is nonlinear (blue dots). There are three replicate measurements for each dilution factor. Our algorithm identifies two linear segments (boundaries marked as circles). Lighter orange circles bound the segment with the highest *R*^2^. We specify the likely maximal range of OD as our prior: [0,2]. Inset: the logarithm of the model evidence for the number of segments. (B) Identifying contiguous linear segments in the logarithm of the OD of growing *E.coli* cells as a function of time allows us to identify automatically the region of exponential growth. We show the mean of four replicate measurements (blue) with twice their standard deviation shaded. Circles denote the boundaries of linear segments; orange circles bound the segment with the best-fit line with highest gradient and so highest specific growth rate. The average specific growth rate over this segment is 1.5 h^–1^. Inset: the logarithm of the model evidence for the number of segments. (C) With our algorithm, we can automatically identify the region of exponential growth in multiple datasets, here 38, to reveal growth laws such as Monod’s equation. We plot the specific growth rate in log phase for *S.cerevisiae* as a function of the concentration of fructose, with the solid line a fit of Monod’s equation: $\lambda_{\text{max}}=0.422\pm 0.006$ h^–1^ and $K_{M}=0.026\pm 0.002$% (w/v). The shaded area shows the 95% confidence interval. Inset: three example growth curves with dots marking the region of exponential growth, identified as the segment with the highest gradient. For panels (B) and (C), we specify a prior on the range of the gradient: [0,5] h^–1^.

The OD of a microbial culture increases linearly with the number of cells only for sufficiently small ODs. At higher ODs, the light from the spectrophotometer may scatter off multiple cells, and the relationship between OD and the number of cells becomes nonlinear (Stevenson *et al.* 2016). To calibrate OD measurements, researchers often serially dilute a dense culture of microbes and measure the relationship between the OD and the dilution factor (Warringer and Blomberg 2003, Stevenson *et al.* 2016) (Fig. 3A). Interpolating this curve, we can convert an OD measurement to the corresponding dilution factor and so correct for any nonlinearity between the OD and cell numbers.

Dilution factors, however, are not intuitive units, and it is useful to identify the range of ODs over which there is a linear relationship with cell numbers. Not only is this range itself important, but by using the ratio of the maximum of the range to the corresponding dilution factor, we can re-scale the dilution factors back into ODs.

We used the nunchaku algorithm to identify the linear range, using basis functions that generate straight lines and an unknown measurement error. Two linear segments are optimal, and the one of interest, where OD is proportional to the number of cells, is the segment beginning at the smallest OD. This segment also has the highest coefficient of determination *R*^2^. Its maximal OD is 0.66 for a relative cell number of 0.25 (Fig. 3A), and we should therefore multiply the dilution factors by 0.66/0.25, or 2.6, to convert back to ODs.

### 3.4 Application 2: identifying the log phase of microbial growth

Microbes are most often studied when growing exponentially, with the log(OD) of the culture increasing linearly with time (Monod 1949). Researchers identify this log-phase growth from microbial growth curves.

To detect log phase automatically, we applied nunchaku, again with basis functions generating lines, to OD measurements of *E.coli* (Fig. 3B). Partitioning the data into six segments is optimal, and the segment whose best-fit line has the highest gradient—the greatest specific growth rate—corresponds to exponential growth.

Monod noticed an empirical relationship between the nutrient concentration and the specific growth rate of microbes in log phase (Monod 1949). Denoting this growth rate as *λ*, the maximal specific growth rate as $\lambda_{\text{max}}$, and the nutrient concentration as *s*, his equation becomes


(28) $$\lambda =\lambda_{\text{max}}\frac{s}{K_{M}+s}$$

where *K_M_* is now called the Monod constant. To estimate $\lambda_{\text{max}}$ and *K_M_*, researchers systematically vary the concentration of the carbon source and identify the log phase and the corresponding gradient for each growth curve.

Here, we use the nunchaku algorithm to select data to estimate $\lambda_{\text{max}}$ and *K_M_* for *S.cerevisiae* growing on fructose (Section 2), from 38 growth curves measured with plate readers (Fig. 3C). Each biological replicate has two technical replicates.

## 4 Discussion

Determining where data are best described by a line is a problem familiar to most scientists. We present a statistically rigorous solution, which we generalize by considering linear combinations of arbitrary basis functions. Our methodology is Bayesian and similar in approach to earlier work that focused on piece-wise constant functions (Hutter 2007).

Like all Bayesian inference, our algorithm depends on prior information: the bounds on the coefficients constituting the linear combination of basis functions. For basis functions generating lines, these bounds describe the range of the gradients and intercepts of all possible lines within a segment. The optimal number of segments will depend on this prior if the amount of data is sufficiently small, as it should (MacKay 2003). In practice, however, users interested in lines need specify only one prior range with the other inferred (Section 2), and we see that although a wide prior favours fewer segments, a single segment is robustly assigned to sections of the data that appear linear.

Our method makes two assumptions about how the data deviate from a linear combination of basis functions. We assume these deviations are independent and we assume that each deviation obeys a normal distribution. For some data, a distribution with a purely nonnegative support, such as a log normal, may be more appropriate. Although we can use such a distribution in principle, in practice some of the steps that we performed analytically would have to become numerical. Further, if nothing is known *a priori* about these deviations, we assume that their standard deviation is identical for all time points. Our algorithm would work too if the standard deviations vary but are proportional to a known function of *x_j_* and *y_j_*.

Our work adds to existing algorithms for detecting change points in time series, including those aimed at analyzing microbial growth (Papastamoulis *et al.* 2019). We have simplified this problem by considering change points to occur only at data points and by imposing no continuity between the functions underlying the data for each segment. These simplifications are not restrictive for our task of finding one particular segment of interest. Identifying change points more generally typically requires Markov chain Monte Carlo methods (Stephens 1994, Papastamoulis *et al.* 2019).

The nunchaku algorithm by using enumeration is robust and lends itself to automation, facilitating high throughput studies. It should both ease and increase the reproducibility of data analyses for a wide range of scientists.

## Supplementary Material

btad688_Supplementary_Data

## Data Availability

The data underlying this article are available in Edinburgh DataShare at https://doi.org/10.7488/ds/7548.
