# Supplementary material for: Nunchaku: optimally partitioning data into piece-wise contiguous segments
Source: Bioinformatics. 2023 Nov 15;39(12):btad688. doi: 10.1093/bioinformatics/btad688 (PMC10697733; doi:10.1093/bioinformatics/btad688)
Supplement: btad688_Supplementary_Data [file btad688_supplementary_data.pdf]

# Supplementary figures for Nunchaku: Optimally partitioning data into piece-wise contiguous segments

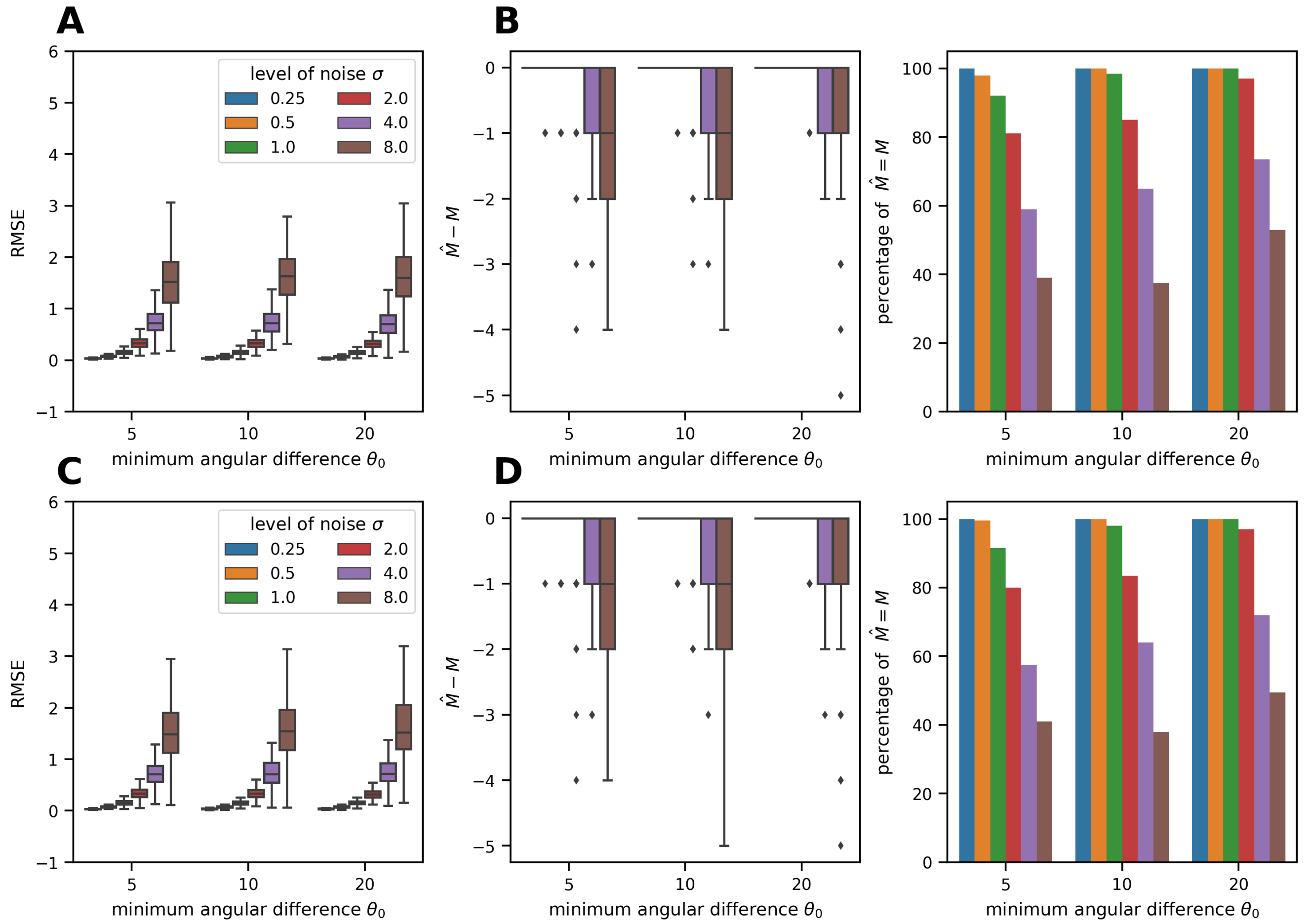


**Figure S1.** The performance of `nunchaku` on synthetic data depends on the minimum angular difference between neighbouring segments $\theta_0$ and the level of noise $\sigma$. **(A, C)** The root mean squared error (RMSE) between the ground truth and the best-fit lines. **(B, D)** The difference between the predicted number of segments $\hat{M}$ and the ground truth $M$ (left) and the percentage of correct predictions of $M$ with $\hat{M} = M$ (right). We generated the data sets following Materials & Methods and ran the analysis with a prior on the range of the gradient: $[-25, 25]$. In panels (A) and (B), we supply `nunchaku` with the true value of $\sigma$ when running the analysis; in panels (C) and (D), the true value of $\sigma$ is not supplied.

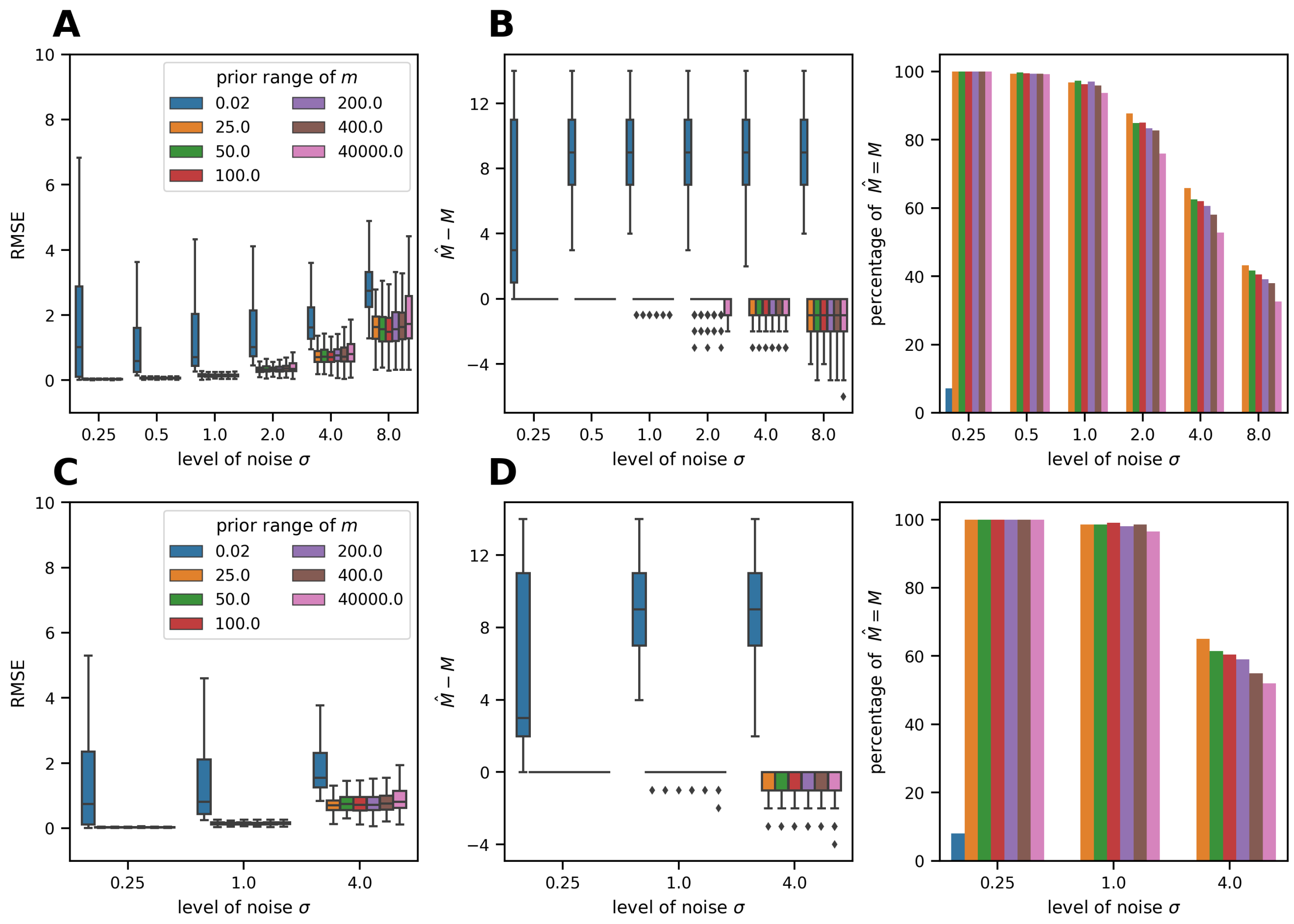


**Figure S2.** The performance of `nunchaku` on synthetic data weakly depends on the *a priori* range of the gradient $m$ when the range is not too small and satisfies Eq. 17. **(A, C)** The root mean squared error (RMSE) between the ground truth and the best-fit lines. **(B, D)** The difference between the number of segments $\hat{M}$ predicted by `nunchaku` and the ground truth $M$ (left) and the percentage of correctly predicting $M$ (i.e. $\hat{M} = M$) (right). We generated the data sets following Materials & Methods and assume a symmetric prior: for example, an *a priori* range of 25 means $[-25, 25]$. The minimum angular difference between neighbouring segments $\theta_0$ is 10°. In panels (A) and (B), we supply `nunchaku` with the true value of $\sigma$ when running the analysis; in panels (C) and (D), the true value of $\sigma$ is not supplied.

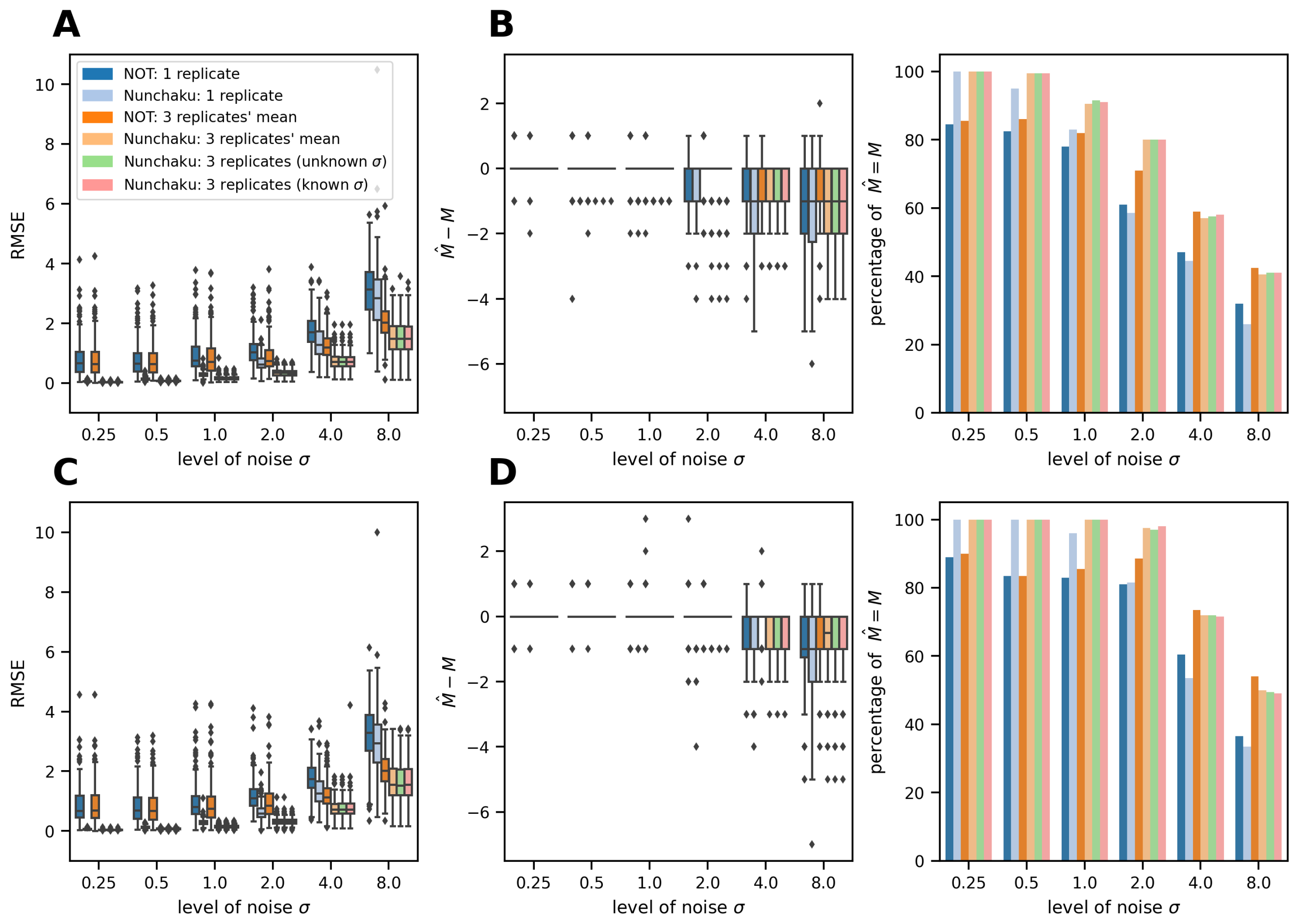


**Figure S3.** The performance of `nunchaku` on synthetic data compared with the Narrowest-Over-Threshold (NOT) algorithm (Baranowski *et al.*, 2019). This algorithm only supports input of one $y$ value for each $x$ value: we therefore input either one replicate or the mean of three replicates. The data is generated similarly to that in Fig. 1 (Materials & Methods). For panels (A) and (B), $\theta_0 = 5°$; for panels (C) and (D), $\theta_0 = 20°$. As a prior for `nunchaku`, we specify only that the gradient of each line lies between $[-25, 25]$. **(A, C)** The root mean squared error (RMSE) between the ground truth and the best-fit lines. **(B, D)** The difference between the predicted number of segments $\hat{M}$ and the ground truth $M$ (left) and the percentage of correct predictions of $M$ with $\hat{M} = M$ (right).

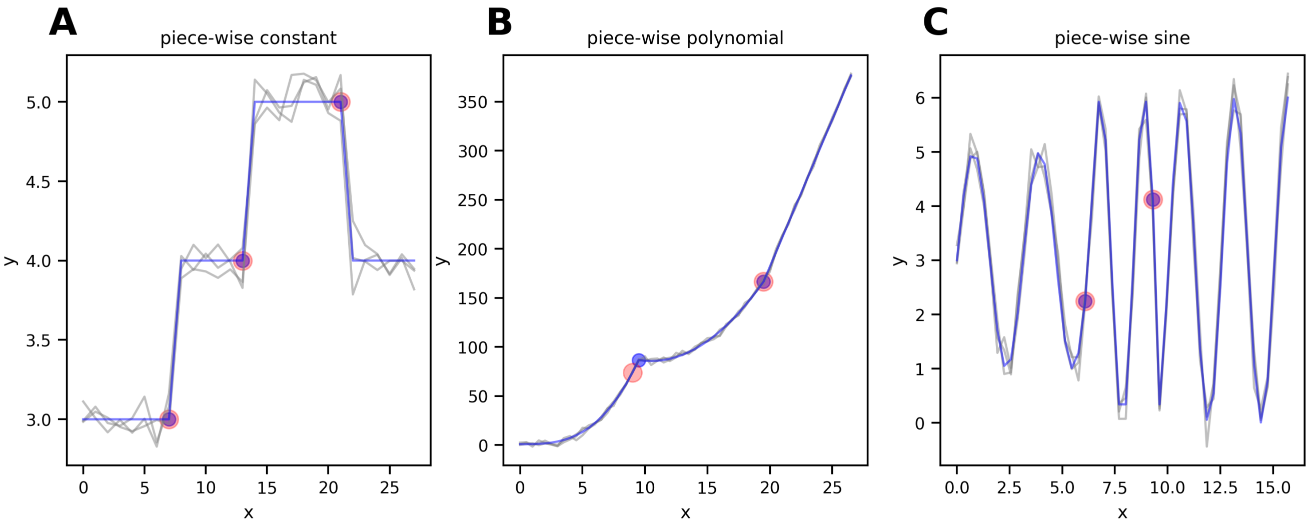


**Figure S4.** The `nunchaku` algorithm correctly predicts the position of the change points in synthetic data generated using either piece-wise constant, polynomial, or sine functions. Each panel is an example synthetic data set with the ground truth in blue and the triplicate data in grey. The red circles are the predicted boundaries. **(A)** A piece-wise constant ground truth. **(B)** A piece-wise polynomial ground truth whose highest degree is three. **(C)** A piece-wise sine function whose amplitude and frequency differ between segments.
